# A School Based Cluster Randomised Health Education Intervention Trial for Improving Knowledge and Attitudes Related to *Taenia solium* Cysticercosis and Taeniasis in Mbulu District, Northern Tanzania

**DOI:** 10.1371/journal.pone.0118541

**Published:** 2015-02-26

**Authors:** Sylvester A. Mwidunda, Hélène Carabin, William B. M. Matuja, Andrea S. Winkler, Helena A. Ngowi

**Affiliations:** 1 Department of Veterinary Medicine and Public Health, Sokoine University of Agriculture, Morogoro, Tanzania; 2 Department of Epidemiology and Biostatistics, College of Public Health, University of Oklahoma Health Sciences Center, Oklahoma City, Oklahoma, United States of America; 3 Department of Neurology, Muhimbili University of Health and Allied Sciences, Dar es Salaam, Tanzania; 4 Department of Neurology, Technical University of Munich, Munich, Germany; Brown University, UNITED STATES

## Abstract

*Taenia solium* causes significant economic and public health impacts in endemic countries. This study determined effectiveness of a health education intervention at improving school children’s knowledge and attitudes related to *T*. *solium* cysticercosis and taeniasis in Tanzania. A cluster randomised controlled health education intervention trial was conducted in 60 schools (30 primary, 30 secondary) in Mbulu district. Baseline data were collected using a structured questionnaire in the 60 schools and group discussions in three other schools. The 60 schools stratified by baseline knowledge were randomised to receive the intervention or serve as control. The health education consisted of an address by a trained teacher, a video show and a leaflet given to each pupil. Two post-intervention re-assessments (immediately and 6 months post-intervention) were conducted in all schools and the third (12 months post-intervention) was conducted in 28 secondary schools. Data were analysed using Bayesian hierarchical log-binomial models for individual knowledge and attitude questions and Bayesian hierarchical linear regression models for scores. The overall score (percentage of correct answers) improved by about 10% in all schools after 6 months, but was slightly lower among secondary schools. Monitoring alone was associated with improvement in scores by about 6%. The intervention was linked to improvements in knowledge regarding taeniasis, porcine cysticercosis, human cysticercosis, epilepsy, the attitude of condemning infected meat but it reduced the attitude of contacting a veterinarian if a pig was found to be infected with cysticercosis. Monitoring alone was linked to an improvement in how best to raise pigs. This study demonstrates the potential value of school children as targets for health messages to control *T*. *solium* cysticercosis and taeniasis in endemic areas. Studies are needed to assess effectiveness of message transmission from children to parents and the general community and their impacts in improving behaviours facilitating disease transmission.

## Introduction

*Taenia solium* is an intestinal and tissue parasite that causes taeniasis and cysticercosis, respectively. The larval stage of *T*. *solium* is found primarily in pigs and the condition is referred to as cysticercosis. Human acquires *T*. *solium* taeniasis through consumption of raw or undercooked pork that contains larvae of this parasite. On the other hand, pigs, humans and a few other animals acquire *T*. *solium* cysticercosis after ingesting viable eggs of this parasite from feeds or water contaminated with human faeces that contain viable *T*. *solium* eggs. Poor hygiene resulting in the contamination of the environment with human faeces put the pigs and people at risk of developing *T*. *solium* cysticercosis [1,2], and is suspected to largely explain the high prevalence of porcine cysticercosis in some developing country areas. If the cysts settle in the human brain or spinal cord they cause neurocysticercosis, with epileptic seizures being the most common symptom [3,4].

Mbulu district of northern Tanzania has been endemic for *T*. *solium* taeniasis-cysticercosis for many years, with an overall district prevalence of porcine cysticercosis reaching 17.4% (n = 770) based on lingual examination [5]. A large village-level randomised trial conducted between 2002 and 2004, estimated an incidence rate of approximately 69 per 100 pig-years based on antigen enzyme-linked immunosorbent assay (Ag-ELISA) [6]. While medical records indicated that epilepsy was an increasing problem in the area as reported by Ngowi [7], Winkler *et al*. [8] confirmed the presence of a strong association between human epilepsy and neurocysticercosis based on a neuroimaging-based study at Haydom Lutheran Hospital, which is situated in Mbulu district. Like in many endemic areas, studies in Mbulu district have observed poor knowledge of affected communities on *T*. *solium* transmission, impact and control [5,6]. The lack of knowledge is presumed to predispose humans to behaviours facilitating transmission of *T*. *solium* infections in humans and pigs.

Previous strategies for control of *T*. *solium* taeniasis and cysticercosis included health education and mass treatment of pigs and humans using anthelmintics [9,10,11]. Nevertheless, to date it is globally not known what intervention strategy really works in the control of *T*. *solium* in an endemic situation [12]. Although health education has been found to be useful in controlling taeniasis and cysticercosis, so far the education has been mostly targeted to adults. Only recently Alexander *et al*. [13] reported improved knowledge and self-reported practices following a health education of school children in India. Nevertheless, because of lack of control group, this study could not estimate the actual effect of the intervention. A randomised controlled trial conducted in Mbulu district of northern Tanzania previously estimated significant improvement of adults’ knowledge and some self-reported practices related to *T*. *solium* [6]. However, this improvement could not be associated with the intervention because of parallel improvement in the control group [6]. Elsewhere, school children have been found to be a good target for messages to control health problems. For example, Lansdown *et al*. [14] advocates health education intervention in school children because they are good knowledge carriers to their respective communities.

The aim of the present study was to determine the effectiveness of health education in improving school children’s knowledge and attitudes related to *T*. *solium* cysticercosis and taeniasis.

## Materials and Methods

### Ethics Statement

The research protocol was approved by the Postgraduate Studies Committee of Sokoine University of Agriculture (the principal author’s institution). This research review board also approved the use of oral consents as requested by the principal author following a pilot study that was conducted in the study area. Before conducting this randomised trial, permission was obtained from Mbulu District Executive Director, District primary and secondary school educational officers and heads of all the 60 participating schools. A parent meeting was organised by each school to inform the parents and guardians on the research and obtain their oral consents for their children to participate. Oral consents were preferred because some of the parents and guardians were not able to read and write. Although only approximately 9.1% of the 2,350 pupils were adults (≥ 18 years old), consents were obtained from all the pupils. The informed consents were recorded in an Excel spread-sheet. After the end of the intervention trial, pupils in the control groups were offered the health education.

### Study area

This study was conducted in Mbulu District located in north-eastern Tanzania, between latitude 3.80^°^S and 4.50^°^S and longitude 35.00^°^E and 36.00^°^E. The major economic activity in the district was crop and livestock production. In 2012, the district had 320,279 people and 70,834 pigs [15]. At the time of this study (2010–2012), the district had a total of 124 primary and 32 secondary schools. Mbulu District was selected for this study because of high endemicity for porcine cysticercosis and low baseline knowledge of farmers on *T*. *solium* during previous studies conducted between 1998 and 2004 [5,6].

### Study design

This was a cluster randomised controlled trial with both pre- and post-intervention assessments of study subjects. The study was conducted from November 2010 to February 2012. Two data collection methods, namely, a questionnaire survey and group discussions were used to support findings.

### Sampling and sample size for the school-based randomized controlled trial

The sample size for the school-based randomized controlled trial was obtained using the World Health Organisation (WHO) multistage cluster sampling formula, which provides a general guidance for use in public health assessments [16]. The method uses a “30 x 7” design which requires a random sample of 30 clusters to be selected from a list of all clusters in the study area, followed by random selection of seven interview sites per cluster. This method is known to enable estimation of prevalence of a factor within +/-10% precision. The variability of the factor decreases when more clusters are used as opposed to when few clusters are used even if the number of interview sites per cluster is increased. In this study, the above method was adopted with slight modification, in which one school grade was purposively selected as an interview site for each school (cluster). In summary, the modified design consisted of random selection of 30 schools (clusters) from each school category (primary, secondary) followed by purposive selection of one grade per school and finally by random selection of one stream per selected grade. A stream consisted of at most 45 pupils (minimum of seven pupils) and all pupils in the selected stream were included in the study. In this context, a stream means a group of pupils of the same grade belonging to one classroom in a school. A particular grade could have several streams depending on the number of pupils registered. Thus our sampling formula was “30 x 1 x 45”. Using this sampling design, the total number of schools initially sampled were 60 (30 primary, 30 secondary) and the expected number of children was 2700 (1350 primary, 1350 secondary), although the final number was less due to variation in the number of children in the selected streams. Grade Seven (Standard Seven in the Tanzanian context) pupils were chosen from primary schools because they had relatively better understanding as compared to lower levels. This is the final-year grade in the primary school education. For secondary schools, Form Two pupils were selected because most schools in the district were still new and had only up to this level of study. The school was the primary unit of sampling and intervention. The use of one interview site per cluster enabled us to minimise intra-cluster correlation, and hence, maximize the information obtained from each cluster. The eligibility criteria for a school was the willingness of the heads of each school to participate and not having participated in the pilot study. There were no exclusion criteria.

### Sampling and sample size for group discussions

Four schools (two primary, two secondary) not involved in school-based randomized trial were selected for the group discussions. The two primary schools were randomly selected from the 94 schools that had not been randomly selected for the school-based randomized trial. The two secondary schools involved in the group discussions were the only two remaining after randomly selecting the 30 secondary schools for the randomized trial. One stream of 45 children was purposively selected from each school. Group discussions were conducted rather than focus group discussions because it was impractical to select a sub-sample of pupils from the class. We thus included all 45 pupils in each selected stream to increase the respondents’ freedom of speech during the discussions. Rabiee [17] advocates the use of pre-existing group as participants become more open to each other and provide true responses. Because of this openness, any insincere response coming up during the discussion is immediately challenged within a group.

### Collection of quantitative baseline data

The baseline component of the randomized trial took place in November 2010. Data collection tools included a self-administered questionnaire. The questionnaire was self-administered in the classroom to each of the participants of the 60 schools and took at most one hour to complete.

The questionnaire contained 37 open and closed-ended questions, including questions assessing pupils’ knowledge on human tapeworm infection (awareness, presence, how acquired, source of knowledge), porcine cysticercosis, human cysticercosis and epilepsy. In addition, two questions examined pupil’s attitudes towards infected pig and pig meat. The questionnaire originally created in English was translated to Kiswahili, the national language. A pilot study was carried out in one school not included in the trial to pre-test the questionnaire before the actual study.

### Collection of qualitative baseline data

Group discussions were conducted two months after the baseline questionnaire had been administered. During this time, only three schools (two primary, one secondary) intended for group discussions were available. A guide was developed and used to guide the discussions. The discussions were conducted in Kiswahili and moderated by the first author (SAM). The discussions were recorded in a notebook. Note that the group discussions were only conducted during the baseline component of the trial. The discussions assessed similar aspects to those in the questionnaire survey for triangulation purpose.

### Randomisation of schools to intervention and control groups

For each of the 30 primary and 30 secondary schools involved in the baseline component of the trial, we first calculated average score on three important knowledge questions (how a person acquires tapeworm infection, if human can be infected with cysticercosis and how a human acquires cysticercosis due to *T*. *solium*). Then the schools were stratified by category and their average scores. Within each school category, the first stratum consisted of schools which scored on average of ≥ 50%. The second stratum involved schools with scores of < 50%. Schools in each stratum were randomly allocated into either intervention or as a control. This resulted into two groups of 15 schools each. All the 30 schools allocated to the intervention group (15 primary, 15 secondary) received the same intervention. One primary school randomised to serve as control mistakenly received the intervention.

### Training of trainers

A one-day training was conducted to update school teachers on issues regarding *T*. *solium* cysticercosis and taeniasis. This was one week before teaching session for school children started. A total of 25 teachers from the intervention groups (13 from primary schools, 12 from secondary schools) were trained. Five schools (two primary, three secondary) did not send their teachers for the training as they lacked science teachers, which was the target for the study. We preferred science teachers to minimise the training time as they have basic science knowledge, and hence, are more likely to easily and correctly grasp the concepts of *T*. *solium* taeniasis and cysticercosis than teachers specialised in art subjects.

### Education of pupils

In March 2011 (three months after the baseline study), intervention groups in both primary and secondary schools were given a health education on cysticercosis and taeniasis by the previously trained teachers. The first author was available during the training and provided clarification where necessary. In addition, this author trained the pupils in the five schools that had no trained teachers (i.e. the five schools where none of the teachers was trained). The training programme included (1) an address by the trainer (2) video show and (3) distribution of leaflets to each participant. Information provided included life cycle of *T*. *solium*, diseases caused by the parasite, mode of transmission, public health and economic impacts as well as prevention and control. The address by the trainer lasted 30 minutes on average. The video was shown in Kiswahili (the national language) followed by Iraqw (the common local language), each of which lasted for 14 minutes.

### Quantitative post-intervention assessments of knowledge and attitudes related to *Taenia solium*

**Fig 1:**
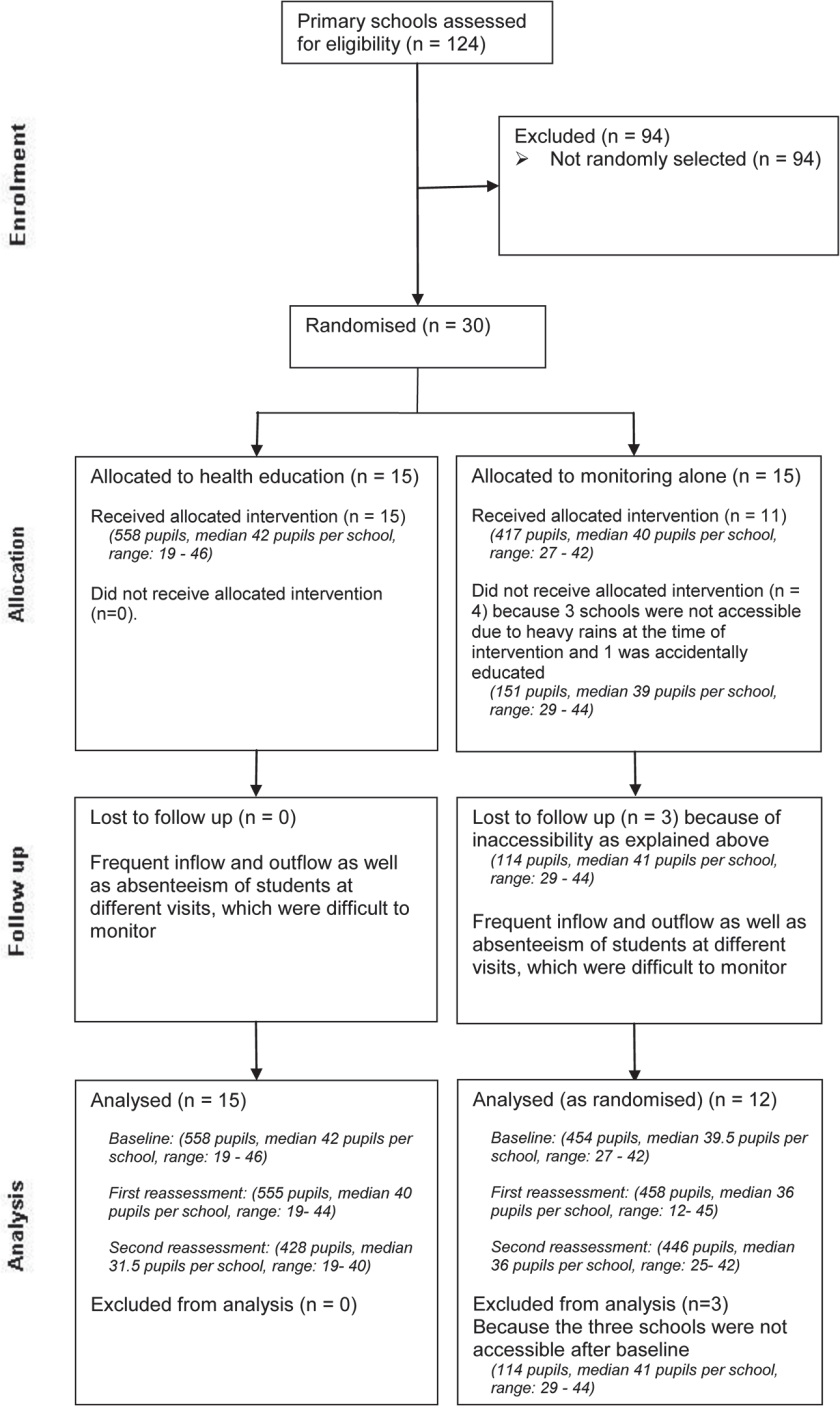
Flow of primary school participants during a cluster randomised health-education intervention trial in Mbulu District, Tanzania, 2010–2012. A random sample of 30 primary schools (1126 students) was randomised into either an intervention (health education) or control (monitoring alone) group and reassessed twice after the health education. During the intervention together with second reassessment, three schools from the control group were not accessible due to heavy rains. In addition, one school from the control group was accidentally educated. Throughout the follow up, there were some losses of students for various reasons. This flow diagram is based on initial randomisation, which resulted into 27 schools analysed.

**Fig 2:**
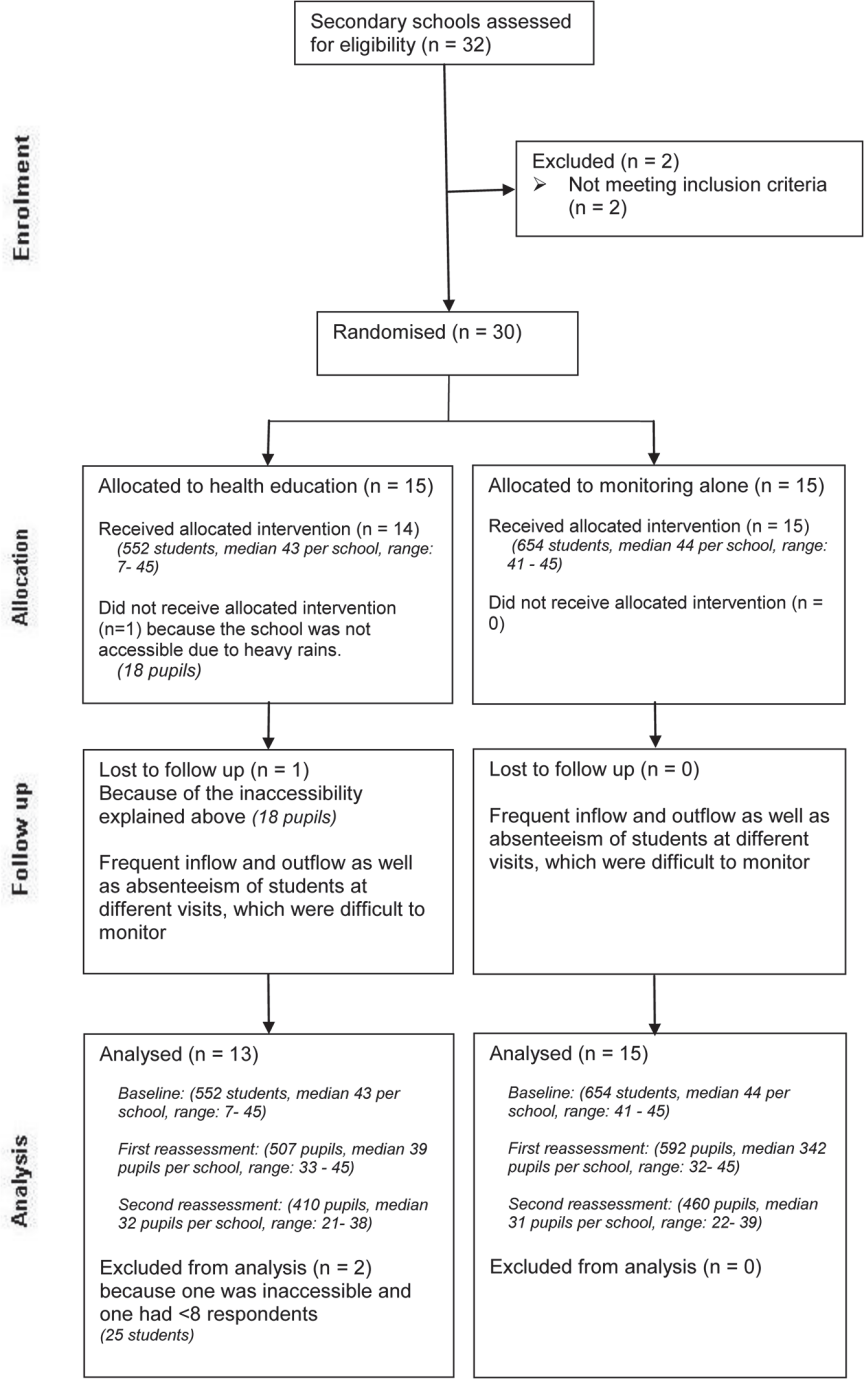
Flow of secondary school participants during a cluster randomised health-education intervention trial in Mbulu District, Tanzania, 2010–2012. A random sample of 30 secondary schools (1224 students) was randomised into either an intervention (health education) or control (monitoring alone) group and reassessed three times after the health education. During the intervention together with second reassessment, one school from the intervention group was not accessible due to heavy rains and one had fewer than 8 pupils. Throughout the follow up, there were some losses of students for various reasons. Thus a total of 28 schools were analysed.

The questionnaire used in the baseline study was used for the post-intervention assessments. The first reassessment was carried out in all schools in March 2011 immediately after the training session conducted in the intervention schools. A total of 56 schools were included. Four schools (three control and one intervention schools) were excluded as it was impossible to access them because of heavy rains. A second reassessment was carried out in August 2011, six months after the intervention in the 56 schools. The third (last) reassessment was done in February 2012, 12 months after the intervention and included only 29 secondary school pupils because primary school pupils had already graduated. Data from one secondary school was excluded from all analyses as less than 8 children answered the questionnaire at each visit. Figs. 1 and 2 present the Consolidated Standards of Reporting Trials (CONSORT) diagram for primary and secondary schools, respectively.

## Statistical analysis

### Quantitative assessment of baseline knowledge and attitudes related to *Taenia solium* infections

Questionnaire data were entered in Microsoft Access 2007 and analysed in Stata 8.0 for Windows. The percentage of correct answers to each question was calculated as the number of pupils providing correct answers to each question divided by the total number of pupils responding to that question in each school. If more than 50% of pupils answered correctly, the knowledge (or attitude) about this question was considered high. We compared the median percentage of correct answers to each question between primary and secondary schools using Wilcoxon rank-sum test. The responses were considered significantly different if the p-value was less than 0.01 to adjust for multiple comparisons. The data at baseline is presented for those schools with at least one follow-up visit and where at least 8 pupils answered the questionnaire (27 primary schools and 28 secondary schools) and according to the reception of the intervention (i.e. efficacy measure).

### Analysis of group discussion data

The three group discussion transcripts were entered into Microsoft Word and analysed in ATLAS.ti 6.2 using the Krueger’s framework [18]. A response was considered as a theme idea if at least two of the three schools mentioned it.

### Analysis of effects of health education on knowledge and attitudes related to *Taenia solium* infections

The data were analysed using the intervention as allocated and as actually received to provide estimates of both effectiveness and efficacy of the intervention, respectively. The school-level effectiveness or efficacy of the intervention and the effect of monitoring (i.e. Hawthorne), adjusted for individual-level age and gender, on improving knowledge and attitudes towards cysticercosis-taeniasis were estimated using Bayesian hierarchical log-binomial models [19]. Five key knowledge variables (heard about porcine cysticercosis, human cysticercosis, epilepsy, human taeniasis and how pigs should be kept) and two attitude variables (would report to a veterinarian if cysts were found under the tongue of a pig and would condemn infected meat) were used as outcomes. The models included the age group and sex of pupils as fixed-effects at the individual level, a random-effect intercept at the school level, and the allocated intervention and timing of the questionnaire as fixed-effects at the school level. For each outcome, three models were run to compare the baseline to the first, second and third re-assessments, with only secondary schools included in the latter.

The overall performance of each pupil on the questionnaire was evaluated by summing all correct answers and dividing them by the total number of questions answered. Fifteen knowledge questions and two attitude questions were included in the overall score. A separate score, which only included the knowledge questions was also calculated. The scores were analysed in Bayesian hierarchical linear regression models using the same hierarchy as for the log-binomial models.

WinBugs software (version 1.4.3, MRC Biostatistics Unit, Cambridge, UK) was used to implement the Gibbs sampler algorithm. Posterior medians of random samples derived from marginal posterior densities were used as point estimates, reported with 95% Bayesian Credible Intervals (95% BCI). The regression coefficients of the log-binomial models were exponentiated to obtain Prevalence Proportion Ratios (PPR). The regression coefficients of the linear models were used to evaluate the average increase in percentage score for each variable of interest. Each model was run with two chains with at least 40,000 iterations. The programmes written in WinBUGS are available upon request to the authors.

## Results

### General characteristics of the study population

The characteristics of the study populations during the baseline questionnaire survey according to the intervention as received and among schools followed-up are shown in Table 1. Intervention and control groups were similar in their baseline demographic characteristics. There was however gradual losses to follow up of schools and pupils for various reasons as described in the CONSORT statements (Fig. 1 and Fig. 2). All the five schools lost to follow up had baseline scores similar to the majority of the analysed schools, suggesting little selection bias.

**Table 1:** Baseline demographic characteristics of children aged 12 to 23 attending primary and secondary schools in Mbulu District, Tanzania, who participated in at least two re-assessments of knowledge and attitude following randomisation of a health education programme.

	Intervention n = 1103	Control n = 1108
***Individual level***		
**Mean (SD) age in years**	15.0 (1.7)	15.1 (1.8)
**Number of Females (%)**	637 (57.8)	637 (57.5)
***School level***		
**Number of primary schools (%)**	15 (53.6%)	12 (44.4%)
**Number of secondary schools (%)**	13 (46.4%)	15 (55.6%)
**Mean (SD) number of children per school**	39.4 (7.81)	41.0 (4.3)

The intervention and control grouping are based on the intervention as randomised and not received (one primary school mistakenly received the intervention).

A total of 135 pupils participated in the three schools included in the group discussions

### Baseline knowledge and attitudes related to *Taenia solium* taeniasis and cysticercosis

The pupils’ baseline knowledge and attitudes related to *T*. *solium* transmission is summarized in Table 2. The intervention and control groups were similar in their baseline average knowledge and attitudes related to epilepsy, taeniasis and cysticercosis, ranging from 0.1–94.6% and 0.2–92.1% in the intervention and control groups, respectively.

**Table 2:** Percentage of correct answers on knowledge and attitudes regarding *Taenia solium* life cycle at baseline in children aged 12 to 23 attending primary (n = 27) and secondary (n = 28) schools in Mbulu District, Tanzania.

	Intervention (I) n = 1103	Control (C) n = 1108
**Knowledge**		
**Has seen or heard about human tapeworm (n**_**I**_ **= 1103, n**_**C**_ **= 1107)**	58.7 (55.8,61.7)	53.0 (50.1,56.0)
**Has seen or heard about porcine cysticercosis (n**_**I**_ **= 1103, n**_**C**_ **= 1107)**	94.6 (93.2, 95.9)	92.1 (90.5, 93.6)
**Has heard about epilepsy (n**_**I**_ **= 1103, n**_**C**_ **= 1105)**	76.3 (73.8, 78.8)	78.2 (75.8, 80.6)
**Best ways of keeping pigs (confinement) (n**_**I**_ **= 1102, n**_**C**_ **= 1107)**	80.9 (78.5, 83.2)	81.7 (79.4, 83.9)
**How a pig acquires cysticercosis (eating human faeces) (n**_**I**_ **= 1043, n**_**C**_ **= 1020)**	74.8 (72.1, 77.4)	70.9 (68.1, 73.7)
**How to recognize an infected pig (tongue/eyelid nodules) (n**_**I**_ **= 1048, n**_**C**_ **= 1019)**	88.7 (86.5, 91.0)	85.5 (83.3, 87.6)
**How to prevent porcine cysticercosis (avoid human faeces) (n**_**I**_ **= 1045, n**_**C**_ **= 1019)**	82.9 (80.6, 85.2)	79.3 (76.8, 81.8)
**Important symptoms of human epilepsy (seizures) (n**_**I**_ **= 843, n**_**C**_ **= 866)**	80.0 (77.2, 82.7)	80.0 (77.4, 82.7)
**How to avoid tapeworm infection (avoid eating raw meat) (n**_**I**_ **= 650, n**_**C**_ **= 588)**	90.5 (88.2, 92.7)	89.1 (86.6, 91.6)
**Human can acquire cysticercosis (n**_**I**_ **= 1045, n**_**C**_ **= 1018)**	33.5 (30.6, 36.4)	34.0 (31.1, 36.9)
**How human acquires cysticercosis (n**_**I**_ **= 352, n**_**C**_ **= 350)**	27.0 (22.3, 31.6)	25.4 (20.8, 30.0)
**Possible cyst locations in human body (to select all—muscles, under skin, brain, eyes) (n**_**I**_ **= 1044, n**_**C**_ **= 1019)**	8.6 (6.9, 10.3)	8.0 (6.4, 9.7)
**Listing cysticercosis as one of epilepsy causes (n**_**I**_ **= 845, n**_**C**_ **= 866)**	0.1 (-0.1, 0.3)	0.2 (1.0, 2.9)
**Attitudes**		
**Would report to a veterinarian if cysticercosis was found in his pig (n**_**I**_ **= 1100, n**_**C**_ **= 1107)**	44.0 (41.1, 46.9)	46.2 (43.2, 49.1)
**Would condemn meat of pig infected with cysticercosis (n**_**I**_ **= 1101, n**_**C**_ **= 1106)**	60.3 (57.4, 63.2)	61.9 (59.1, 64.8)

Most schools showed low knowledge (<50% pupils answering correctly) regarding human cysticercosis. On the other hand children had high baseline knowledge on epilepsy, taeniasis and porcine cysticercosis. High or low scores by a school in a particular question did not necessarily predict similar levels of performance in other questions.

### Results of group discussions

Results from group discussions were similar to those of the questionnaire survey as most themes that emerged from the discussions matched with the most common answers given in response to the questions asked during the questionnaire survey. When asked on how a human acquires taeniasis, a variety of answers were given. Consumption of raw or inadequately cooked meat in general emerged as a theme idea. One of the three groups was able to cite measly pork as a cause of taeniasis in human. A common perception on the cause of human cysticercosis was consumption of measly pork. Some scattered ideas were raised such as drinking of un-boiled water, eating foods with dirty hands or eating unwashed fruits or vegetables. Nevertheless, no group mentioned human faeces as a source of cysticercosis. Participants had good knowledge of epilepsy symptoms, and seizures, falling down accompanied with loss of consciousness and froths from the mouth emerged as theme ideas. When asked on the causes of epilepsy in humans, two theme ideas were raised, namely, witchcraft and infection of the brain with worm or malaria parasites. Two theme ideas emerged with regard to how a pig could acquire cysticercosis. Group discussion participants believed that a pig could acquire cysticercosis through consumption of human faeces, which the authors considered correct at this participant’s level of understanding. It was also commonly believed that a pig could acquire cysticercosis through consumption of pig products such as meat, bones, or milk from a pig infected with cysticercosis. All the groups cited white nodules as common indicators of cysticercosis in pigs. Keeping pigs indoors was a strongly believed measure to prevent pigs from acquiring cysticercosis. Regarding measures taken when a live pig was recognized to be infected with cysticercosis, two theme ideas emerged, namely, consult with a veterinarian or use traditional medicine. On the other hand, when pork for home consumption was recognised to be infected with cysticercosis, some people would cook it thoroughly and consume it while others would dispose it.

### Efficacy and effectiveness of health education intervention on knowledge and attitudes regarding *Taenia solium* infections

Table 3 presents school-level efficacy of the intervention and the effect of monitoring alone on correct answers on five knowledge and two attitudes questions regarding *T*. *solium* life cycle for all schools. The intervention improved knowledge about human cysticercosis the most, followed by that about human tapeworm, epilepsy and porcine cysticercosis. The intervention did not, however, improve knowledge on how to best keep pigs, while monitoring alone slightly improved it. The knowledge improvement persisted throughout the six months of evaluation, although the improvement in knowledge about human cysticercosis was somewhat lower at 6 months. While the attitude to condemn infected pork was improved significantly by the intervention, the children’s willingness to report an infected pig to a veterinarian was considerably reduced by the intervention. Similar trends were observed when secondary schools only were analysed (Table 4), which also allowed for an assessment of the 12-months efficacy of the intervention. As observed in all schools, the efficacy of the intervention at improving knowledge about human cysticercosis slightly decreased through time, but the effect of monitoring alone improved, suggesting that some knowledge may have been shared among schools in the longer term. The results for knowledge about porcine cysticercosis were less conclusive due to the instability of the model created by the very high initial knowledge observed. Unlike what was found when all schools were analysed, knowledge about the best way of keeping pigs was improved up to 6 months post intervention in secondary schools, but not after 12 months. Monitoring alone increasingly improved this knowledge through time, again suggesting communication among pupils from different schools. These results suggest that information may be shared more easily among older pupils attending secondary schools than pupils attending primary schools.

**Table 3:** Prevalence Proportion Ratios (PPR) and 95% Bayesian Credible Intervals (95%BCI) of the school-level effects of the intervention and monitoring on correct answers on knowledge and attitudes regarding *Taenia solium* life cycle in children aged 12 to 23 attending primary (n = 27) and secondary (n = 28) schools in Mbulu District, Tanzania.

	Baseline to visit 1		Baseline to visit 2	
	Intervention effect	Monitoring effect	Intervention effect	Monitoring effect
**Knowledge**				
**Has seen or heard about human tapeworm**	1.69 (1.54–1.87)	1.18 (1.11–1.27)	1.69 (1.55–1.84)	1.04 (0.97–1.11)
**Has seen or heard about porcine cysticercosis**	1.10 (1.06–1.15)	1.01 (0.99–1.03)	1.11 (1.07–1.17)	1.00 (0.97–1.02)
**Has heard about human cysticercosis**	3.16 (2.79–3.62)	0.95 (0.85–1.07)	2.42 (2.15–2.76)	1.04 (0.92–1.16)
**Has heard about epilepsy**	1.30 (1.22–1.38)	1.03 (0.99–1.07)	1.24 (1.71–1.31)	0.95 (0.91–0.99)
**Best ways of keeping pigs (confinement)**	0.96 (0.92–1.01)	1.14 (1.11–1.18)	0.99 (0.96–1.03)	1.12 (1.08–1.16)
**Attitudes**				
**Would report to a veterinarian if cysticercosis was found in his pig**	0.54 (0.47–0.62)	1.04 (0.96–1.13)	0.56 (0.48–0.66)	0.94 (0.85–1.03)
**Would condemn meat of pig infected with cysticercosis**	1.10 (1.02–1.19)	1.03 (0.97–1.09)	1.24 (1.14–1.35)	0.94 (0.88–1.01)

All PPR are adjusted for the age and gender of respondents and the clustering effect of the school using a Bayesian hierarchical log-binomial model.

**Table 4:** Prevalence Proportion Ratios (PPR) and 95% Bayesian Credible Intervals (95%BCI) of the school-level effects of the intervention and monitoring on correct answers on knowledge and attitudes regarding *Taenia solium* life cycle in children aged 13 to 23 attending secondary schools (n = 28) in Mbulu District, Tanzania.

	Baseline to visit 1		Baseline to visit 2		Baseline to visit 3	
	Intervention effect	Monitoring effect	Intervention effect	Monitoring effect	Intervention effect	Monitoring effect
**Knowledge**						
**Has seen or heard about human tapeworm**	1.31 (1.17–1.47)	1.19 (1.09–1.29)	1.47 (1.32–1.63)	1.10 (1.01–1.21)	1.50 (1.35–1.68)	0.98 (0.89–1.08)
**Has seen or heard about porcine cysticercosis**	31.06 (1.56–908.69)	1.00 (0.97–1.04)	1.17 (1.09–1.29)	0.98 (0.94–1.01)	0.97 (0.92–1.00)	1.01 (0.98–1.05)
**Has heard about human cysticercosis**	2.44 (2.08–2.86)	1.03 (0.90–1.18)	1.92 (1.66–2.23)	1.13 (0.98–1.30)	1.54 (1.29–1.84)	1.24 (1.06–1.44)
**Has heard about epilepsy**	1.22 (1.13–1.31)	0.97 (0.92–1.01)	1.19 (1.11–1.28)	0.93 (0.87–0.98)	1.27 (1.17–1.38)	0.91 (0.86–0.97)
**Best ways of keeping pigs (confinement) (variable is BestWaysOfKeepingPigs)**	1.12 (1.07–1.17)	1.024 (1.00–1.08)	1.06 (1.03–1.11)	1.05 (1.00–1.08)	0.99 (0.95–1.03)	1.08 (1.04–1.12)
**Attitudes**						
**Would report to a veterinarian if cysticercosis was found in his pig**	0.54 (0.46–0.64)	1.08 (0.98–1.19)	0.56 (0.48–0.66)	0.94 (0.85–1.03)	0.59 (0.49–0.741)	1.12 (1.00–1.26)
**Would condemn meat of pig infected with cysticercosis**	1.27 (1.14–1.41)	0.97 (0.89–1.05)	1.38 (1.22–1.55)	0.89 (0.81–0.98)	1.37 (1.21–1.55)	0.90 (0.81–1.00)

All PPR are adjusted for the age and gender of respondents and the clustering effect of the school using a Bayesian hierarchical log-binomial model.

Table 5 presents school-level efficacy of the intervention and the effect of monitoring alone on the overall score and knowledge score in all the studied schools. The intervention and monitoring alone improved the knowledge scores by an average of about 10–11% and 6–7%, respectively, and these were sustained throughout the six months follow-up. The intervention improved the total score by about 9–10%. However, while monitoring alone did improve the overall score by an average of 6.5% (95%BCI: 5.5%-7.5%) immediately after the intervention, it decreased to 3.2% (95%BCI: 2.2%-4.3%) after 6 months. When secondary schools (followed for 12 months) were analysed alone, the intervention improved the knowledge and total scores by approximately 7–8% sustainably over 12 months (Table 6). Monitoring alone led to similar levels and trend of knowledge improvement (in the order of 6–7%), which further supports our suggestion that secondary school pupils may communicate among schools. The lack of improvement of attitudes by monitoring alone led to its effect on total score being lower.

**Table 5:** Coefficients of regression and 95% Bayesian Credible Intervals (95%BCI) of the school-level effects of the intervention and monitoring on the proportion of correct answers to knowledge and knowledge and attitudes questions regarding *Taenia solium* life cycle in children aged 12 to 23 attending primary (n = 27) and secondary (n = 28) schools in Mbulu District, Tanzania.

	**Baseline to visit 1**		**Baseline to visit 2**	
	**Intervention effect**	**Monitoring effect**	**Intervention effect**	**Monitoring effect**
**Knowledge score**	10.9 (9.61–12.16)	7.15 (6.18–8.13)	10.12 (8.77–11.45)	6.32 (5.28–7.36)
**Total score**	8.92 (7.65–10.17)	6.49 (5.53–7.45)	9.63 (8.27–10.97)	3.22 (2.22–4.25)

All regression coefficients are adjusted for the age and gender of respondents and the clustering effect of the school using a Bayesian hierarchical linear model.

**Table 6:** Coefficients of regression and 95% Bayesian Credible Intervals (95%BCI) of the school-level effects of the intervention and monitoring on the proportion of correct answers to knowledge and knowledge and attitudes questions regarding *Taenia solium* life cycle in children aged 13 to 23 attending secondary schools (n = 28) in Mbulu District, Tanzania.

	**Baseline to visit 1**		**Baseline to visit 2**		**Baseline to visit 3**	
	**Intervention effect**	**Monitoring effect**	**Intervention effect**	**Monitoring effect**	**Intervention effect**	**Monitoring effect**
**Knowledge score**	7.80 (5.98–9.60)	7.56 (6.30–8.83)	6.39 (4.42–8.31)	6.94 (5.56–8.33)	6.78 (4.73–8.78)	6.99 (5.50–8.49)
**Total score**	6.47 (4.65–8.26)	6.74 (5.49–8.00)	6.15 (4.21–8.07)	3.92 (2.56–5.28)	6.11(4.04–8.15)	4.68 (3.16–6.20)

All regression coefficients are adjusted for the age and gender of respondents and the clustering effect of the school using a Bayesian hierarchical linear model.

Similar results were obtained when data were analysed using the allocation of the intervention to measure effectiveness. As expected, the effect of the intervention was slightly reduced while that of monitoring was slightly increased. Results of these analyses are available as supplemental material.

### Effect of age and gender on knowledge and attitudes regarding *Taenia solium* infections

While age and gender did not affect knowledge and attitudes for most questions, boys tended to be more likely to report to a veterinarian if cysts were found in a live pig. In contrast, boys were less likely than girls to condemn an infected pork carcass. Boys tended to have more knowledge about human cysticercosis than girls. Older age groups tended to be more willing to condemn an infected pork carcass and to consult a veterinarian if a live pig was found infected. In terms of knowledge and overall scores, boys and older age groups generally performed better.

## Discussion

To the best of our knowledge, this is the first randomised controlled health education intervention trial conducted in school children for assessment of knowledge and attitudes related to *T*. *solium* cysticercosis and taeniasis in an endemic area. It is also the first time retention of knowledge following a health education on *T*. *solium* is assessed. The inclusion of a control group enabled us to estimate better the net effect of the intervention by disentangling possible educational effect of the questionnaire survey itself (i.e. the effect of monitoring alone).

This study found large variations in the baseline knowledge and attitudes regarding *T*. *solium* cysticercosis and teaniasis in several variables tested among schools. The pupils’ knowledge was high in most aspects related to *T*. *solium* lifecycle, especially regarding cysticercosis in pigs and tapeworm infection in humans. On the other hand, the pupils had very low knowledge on all four issues asked in relation to human cysticercosis, namely, susceptibility, mode of transmission, cyst predilection sites and whether cysticercosis could cause epilepsy in human. When comparing the findings obtained using questionnaire and group discussions, it was noted that both methods produced similar results. Only minor differences occurred on the attitudes of children in controlling taeniasis/cysticercosis. While in the questionnaire most pupils showed that they were not ready to consume cysticercotic pork, pupils in group discussions showed their willingness to consume it after thorough cooking. The latter is likely to be the actual behaviour prevailing in the community. Indeed, while people in the group discussions are more likely to tell the truth in the presence of their peers, responses to a questionnaire may reflect “desired” behaviours. A village-level clustered randomised controlled health education intervention trial conducted eight years prior to the current trial in the same area found relatively lower baseline knowledge of farmers than in the current study, though the relative knowledge between the different forms of *T*. *solium* infections and attitude towards consumption of infected pork was similar to the current study [6]. The observed higher baseline knowledge of children than that of farmers in the prior trial could be due to the effect of the previous education in the area, which could have transmitted some to the local children. Although the exposure to previous research and education on *T*. *solium* could have influenced children’s baseline knowledge, this is less likely to have affected the estimation of intervention’s effectiveness as both control and intervention schools belong to the same community. Surprisingly, the attitude towards consumption of infected pork has not changed over time despite the farmers’ education. This could be caused by poverty and/or value attached to pork in the study community.

The present study observed similar levels of baseline knowledge and attitudes among pupils of primary and secondary schools. The similarities in the level of knowledge about *T*. *solium* despite the children’s difference in their level of study might be due to education provided by parents or other community members to children while they are still in primary school. By the time children reach the last year of primary school, they would have acquired similar levels of information.

The intervention was efficacious at improving four of the five knowledge questions and one of two attitude questions, an improvement which was sustained through time and consistent across all and secondary school pupils. Only the knowledge regarding the best way to keep pigs did not improve and the attitude of reporting to a veterinarian if a pig was found infected with cysticercosis was found to be negatively influenced by the intervention. This study assessed only two attitude variables, namely, the action that the respondent would take if she/he recognised cysticercosis in her/his pig and what one would do if pork intended for consumption was found to have cysticercosis, limiting our ability to conclude on the overall attitude of the respondents. However, the observed results can guide further studies and control activities for *T*. *solium*. While the health education improved children’s desire to condemn infected pork, it reduced their willingness to report to a veterinarian if they recognise cysticercosis in their live pigs. Though killing and disposing cysticercotic pigs would be the right measure considering that currently there is no effective treatment available to farmers to treat infected pigs in this area, this response was considered incorrect because the health education recommended consulting a livestock officer when an infected pig was found. Nevertheless, this response suggests possible worries that the children faced having been informed on the human health effects associated with porcine cysticercosis and currently lack of readily available treatment for infected pigs. Such worries support the Health Belief Model (HBM) [20]. The HBM has six postulates and two of them might be the cause for this observation. One of the postulates is the perceived susceptibility to the disease, i.e. how a person feels in case he contracts the disease, but another postulate is the perceived severity of the disease, i.e. how serious will it be if he contracts the disease. Following school children education, the acquired knowledge made the children to relate NCC with epilepsy and nobody really wanted to have epilepsy and therefore they wanted to get rid of the infected pigs to avoid being infected.

The intervention was generally efficacious at improving the knowledge score of all schools pupils, an improvement which persisted through time. Interestingly, monitoring alone also contributed to improving the knowledge and overall scores, although its persistence through time was less marked. The fact that monitoring alone showed improvement in scores immediately after the first training (the same day) shows that the administration of the questionnaire itself may have led to some improvement in knowledge. Being asked the same questions twice in a short interval of time may have led children to think through their answers more carefully. This immediate improvement in scores was observed at a similar magnitude when all schools or secondary schools alone were analysed.

The effect of monitoring on knowledge about human cysticercosis and the best way of keeping pigs as well as the attitude towards reporting to a veterinarian if a pig was to be found infected increased with time among secondary schools, but not when all schools were analysed together. This suggests that secondary schools students may interact among themselves and across schools. Most secondary schools are day schools meaning that children report to school in the morning, study during the day and go back their families at the end of the day. Although primary schools pupils also go home every day, the apparent lower level of interaction among pupils of primary schools may be due their personal interests and roles in the families, which might differ from those of secondary school pupils. At schools, most of primary school pupils would use most of their free time playing games while most of the secondary school pupils would use it for studying or socialisation. At homes, primary school pupils are the most directed by their parents to perform some family duties such as fetching water, fire woods and grazing animals. On the other hand secondary school pupils are more respected, which may give them more time to meet with their peers for socialisation. All these increase chances for higher exchange of information among secondary school pupils.

The present study found that after the health education boys were more likely to consult a veterinarian if their pigs were infected with cysticercosis than girls. In other words, girls would rather kill and dispose of infected pigs. This is likely nature in this rural area where although women are the mostly involved in raising pigs, their role is mainly feeding and general care. Men are more involved in taking care of pig health and likely passed the skills to the young boys. The study also found that increased age was associated with positive attitude to condemn infected pork and consult with a veterinarian for infected pigs as well as better scores. This is most likely because of generally increased understanding on the health risks as the age increases.

The present study had some limitations worth mentioning. The number of children studied declined gradually over time due to various reasons, including absenteeism and occasional transfers from one school to another, which were difficult to monitor during the study. The decline in the number of children together with the exclusion of five schools (four with no follow-up data, one with fewer children) might have influenced the results of this study. However, the fact that these events occurred similarly in the intervention and control groups, their effects on the effectiveness of the intervention are judged to be minimal. Age and gender of respondents at each assessment was adjusted statistically in the models. Combining qualitative and quantitative research methods (triangulation) as done in this study, helps to better understand a phenomenon in context [21]. We also understand that some social desirability bias might have affected the results of our study when trying to measure attitudes. However, the use of “self-administered” questionnaire with anonymity of the respondent might have helped to minimise the bias. Finally, to increase participation, the children were not identified at each assessment, making it impossible to consider non independence of children answering the questions in this study. We also acknowledge a possible limitation in the knowledge and attitude measures used in this research. The relatively few number of items used might have posed limitations to the inferences drawn. Use of a more detailed multi-item measure could provide possibly more nuanced estimation of changes in relevant knowledge and attitudes. The failure of this study to follow up primary schools throughout the 12 months of assessment, limited assessment of the efficacy and effectiveness of this category of schools during the whole period and its comparison with that of secondary schools. However, it is anticipated that the effect of the intervention would have been slightly higher in primary schools because of the observed possible minimal interactions among primary as compared to secondary school children.

Generally, health education conveyed to the pupils of the intervention group improved many aspects of knowledge and attitudes regarding control of *T*. *solium* cysticercosis and taeniasis. Since the improvement was observed to persist in the subsequent reassessments after the intervention, the authors believe that school children will be helpful in controlling the infections in the community. In addition, educating children about cysticercosis and taeniasis is important for self-prevention from infections with the parasites. Nevertheless, more studies are needed to further assess the length of time to which the acquired knowledge would persist as well as its contribution to behaviour change and reduction in disease burden. A study to determine the effectiveness of a health and hygiene education intervention on the occurrence of soil-transmitted helminths re-infection four months post-de-worming found that the intensity of *Ascaris lumbricoides* infection at follow-up was significantly lower (by 58%) in children in intervention schools compared with children in control schools [22].

## Supporting Information

S1 File Data. (XLS)

S1 Table Effectiveness of the school based health education intervention. Data were analysed as per the intervention allocation in which one accidentally educated control school was maintained in the control group.
(DOC)

S1 Text Questionnaire survey. (DOC)
