# Supplementary material for: A School Based Cluster Randomised Health Education Intervention Trial for Improving Knowledge and Attitudes Related to Taenia solium Cysticercosis and Taeniasis in Mbulu District, Northern Tanzania
Source: PLoS One. 2015 Feb 26;10(2):e0118541. doi: 10.1371/journal.pone.0118541 (PMC4342010; doi:10.1371/journal.pone.0118541)
Supplement: S1 Table — Data were analysed as per the intervention allocation in which one accidentally educated control school was maintained in the control group. (DOC) [file pone.0118541.s002.doc]

# Table.S1. Effectiveness of the school based health education intervention. In the current results, data were analysed as per the intervention allocation in which one accidentally educated control school was maintained in the control group. The efficacy of the intervention is presented in the manuscript using intervention as received.

**Table 7. Prevalence Proportion Ratios (PPR) and 95% Bayesian Credible Intervals (95%BCI) of the school-level effects of the intervention and monitoring (as allocated) on correct answers on knowledge and attitudes regarding *Taenia solium*** life cycle in children aged 12 to 23 attending primary (n=27) and secondary (n=28) schools in Mbulu District, Tanzania.

|  | **Baseline to visit 1** | | **Baseline to visit 2** | |
| --- | --- | --- | --- | --- |
|  | **Intervention effect** | **Monitoring effect** | **Intervention effect** | **Monitoring effect** |
| **Knowledge** |  |  |  |  |
| **Has seen or heard about human tapeworm** | 1.59 (1.44-1.77) | 1.22 (1.14-1.31) | 1.60 (1.47-1.74) | 1.07 (1.00-1.15) |
| **Has seen or heard about porcine cysticercosis** | 1.10 (1.05-1.15) | 1.01 (0.99-1.03) | 1.11 (1.07-1.17) | 1.00 (0.98-1.02) |
| **Has heard about human cysticercosis** | 2.83 (2.48-3.22) | 1.06(0.94-1.19) | 2.25 (1.98-2.54) | 1.11 (0.99-1.24) |
| **Has heard about epilepsy** | 1.27 (1.20-1.35) | 1.04 (1.00-1.08) | 1.22 (1.16-1.29) | 0.96 (0.91-1.00) |
| **Best ways of keeping pigs (confinement)** | 0.96 (0.92-1.00) | 1.15 (1.11-1.19) | 0.99 (0.95-1.03) | 1.13 (1.09-1.16) |
| **Attitudes** |  |  |  |  |
| **Would report to a veterinarian if cysticercosis was found in his pig** | 0.55 (0.48-0.63) | 1.03 (0.95-1.12) | 0.58 (0.49-0.67) | 0.92 (0.84-1.01) |
| **Would condemn meat of pig infected with cysticercosis** | 1.08 (1.00-1.17) | 1.04 (0.98-1.11) | 1.23 (1.13-1.33) | 0.96 (0.89-1.02) |

All PPR are adjusted for the age and gender of respondents and the clustering effect of the school using a Bayesian hierarchical log-binomial model.

**Table 8. Coefficients of regression and 95% Bayesian Credible Intervals (95%BCI) of the school-level effects of the intervention and monitoring (as allocated) on the proportion of correct answers to knowledge and knowledge and attitudes questions regarding *Taenia solium* life cycle in children aged 12 to 23 attending primary (n=27) and secondary (n=29) schools in Mbulu District, Tanzania.**

|  | **Baseline to visit 1** | | **Baseline to visit 2** | |
| --- | --- | --- | --- | --- |
|  | **Intervention effect** | **Monitoring effect** | **Intervention effect** | **Monitoring effect** |
| **Knowledge score** | 9.98 (8.69 -11.27) | 7.80 (6.84-8.77) | 9.16 (7.78-10.51) | 6.99 (5.94-8.03) |
| **Total score** | 8.13 (6.85-9.40) | 7.04 (6.10-7.99) | 8.77 (7.39-10.11) | 3.83 (2.80-4.86) |

All regression coefficients are adjusted for the age and gender of respondents and the clustering effect of the school using a Bayesian hierarchical linear model.
