# Supplementary material for: A School Based Cluster Randomised Health Education Intervention Trial for Improving Knowledge and Attitudes Related to Taenia solium Cysticercosis and Taeniasis in Mbulu District, Northern Tanzania
Source: PLoS One. 2015 Feb 26;10(2):e0118541. doi: 10.1371/journal.pone.0118541 (PMC4342010; doi:10.1371/journal.pone.0118541)
Supplement: S1 Text — (DOC) [file pone.0118541.s003.doc]

# Text.S1. Questionnaire survey

QUESTIONNAIRE TO ESTIMATE KNOWLEDGE, ATTITUDES AND PRACTICES OF SCHOOL CHILDREN RELATED TO *TAENIA SOLIUM* INFECTIONS, MBULU DISTRICT, NORTHERN TANZANIA

**General instructions**

- Some questions will require you to fill in the blanks provided
- For multiple choice questions, circle the letter of the correct answer (s)
- Put a **
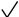
** sign in the box provided for YES or NO questions

**DEMOGRAPHIC INFORMATION OF THE REPSONDENT**

1. Age (years) ...............................
2. Sex ................................
3. Class .............................
4. Name of school.........................................................................
5. District .....................................................................................
6. Region .....................................................................................
7. Date ........................................................................................
8. **KNOWLEDGE ON TAENIOSIS AND CYSTICERCOSIS**
9. Have you ever seen or heard about tapeworm infection in human? (If No, go to question number 7)

Yes No

1. Have you ever heard of anyone having tapeworm infection in this school or elsewhere?

Yes No

1. How can a person acquire tapeworm infection?
2. By consuming raw or inadequately cooked pork or beef
3. By consuming any raw or inadequately cooked meat
4. None of the above is correct
5. All of the above are correct
6. Which among the following are the signs of tapeworm infection in human?
7. Expulsion of tapeworm segment(s) in faeces
8. Fever and presence of skin nodules
9. None of the above is correct
10. All of the above are correct
11. How can a person with tapeworm infection be cured?
12. Using traditional medicine
13. Using modern medicine (go to hospital)
14. None of the above is correct
15. All of the above are correct
16. How can a person avoid tapeworm infection? (circle all correct answers)
17. Eat meat only inspected by a government meat inspector
18. Avoid eating raw or undercooked meat
19. Wash hands frequently
20. All of the above are correct
21. None of the above is correct
22. Have you ever heard of porcine cysticercosis (fini ya nguruwe)? (If No, go to question number 15)

Yes No

1. Where did you first hear about porcine cysticercosis? (circle all correct answers)
2. From my parents or family member
3. From my teacher at school
4. From other sources (specify)............................................................................
5. How can a pig acquire cysticercosis?
6. By eating human faeces
7. By eating animal faeces
8. None of the above is correct
9. All of the above are correct
10. How can you recognize cysticercosis in a live pig? (circle all correct answers)
11. I cannot recognise
12. An infected pig becomes thin
13. Presence of nodules under the eyelids
14. Presence of nodules under the tongue
15. Any other explanation.................................................................................
16. How can one prevent a pig from cysticercosis? (circle all correct answers)
17. Cannot be prevented
18. Use traditional medicine
19. Prevent pigs access to human faeces
20. None of the above is correct
21. Can a human being also be infected with cysticercosis (fini)?
22. Yes
23. No
24. I don’t know
25. If yes, how can a person possibly acquire cysticercosis?
26. By eating raw or undercooked meat
27. By eating foods or drinking water contaminated with human faeces
28. None of the above is correct
29. All of the above are correct.
30. In human, where can cysticerci be located? (choose one most correct)
31. In the muscles
32. Under the skin
33. In the brain
34. In the eyes
35. All of the above are correct
36. Have you ever heard of human epilepsy? (If no go to question number 21)

Yes No

1. Which of the following is among the symptoms of epilepsy in human?
2. Abdominal pain, vomiting and diarrhoea
3. Fever
4. Loss of consciousness and frequent seizures
5. None of the above is correct
6. List down four major causes of epilepsy in human

........................................................................................................................

........................................................................................................................

........................................................................................................................

........................................................................................................................

1. Is there any relationship between pork consumption and epilepsy?

Yes No

1. If “Yes” briefly explain the relationship....................................................................

.....................................................................................................................................

1. How can a person with epilepsy be cured? (circle all correct answers)
2. I don’t know
3. Use modern medicine (go to hospital)
4. Use traditional medicine.
5. Any other explanation.......................................................................................
6. In your studies, is there any subject that teaches you about diseases?

Yes No

ghh

ghh

1. If “ Yes” what is the name of the subject?..................................................................
2. List down all the diseases that you have covered in this subject.....................................

......................................................................................................................................

1. **PRACTICES**
2. Do you keep pigs at home? ................................ (If no go to question 27 )
3. Where do your adult pigs stay during daytime?
4. They roam freely
5. They are tethered outside
6. They are kept indoors
7. Any other explanation..............................................................................
8. Where do your piglets stay during daytime?
9. They roam freely
10. They are tethered outside
11. They are kept indoors
12. Any other explanation..............................................................................
13. Which of the following ways of keeping pigs is the best, and why?
14. Free ranging the pigs all the time
15. Tethering the pigs all the time
16. Housing the pigs all the time
17. Housing the pigs at night and letting them to roam during the day

Reasons for your choice.....................................................................................

1. Do you consume pork?

Yes No

ghh

ghh

1. If “Yes” in what form?
2. Boiled
3. Fried
4. Raw
5. Any other form? Specify.....................................................................................
6. Have you slaughtered or observed slaughtering of a pig at your home?

Yes No

ghh

ghh

1. If “Yes” how did you know whether or not the pork was fit for human consumption?
2. A government meat inspector inspected the meat
3. The meat was not inspected
4. Any other explanation..........................................................................................
5. If you found pork to have cysticerci what would you do with the meat? .......................

......................................................................................................................................

1. What would you do if you discovered that your live pig at home had cysticercosis?

......................................................................................................................................

1. If after receiving a health education you went home and informed your parents about causes, impacts and the right practices to prevent porcine cysticercosis and human tapeworm, how would your parents possibly respond?
2. I do not know
3. They would not listen to me
4. They would listen but would not implement my recommendations
5. They would listen and implement my recommendations
6. Provide reasons for your answer in Question 34 above...................................................

......................................................................................................................................

1. Who among the following children your parents would respect their idea most? (select all correct and number them in the order of priority)
2. Pre-school children
3. Primary school children
4. Secondary school children
5. College or university students
6. Who among the following children your parents would not respect their idea at all? (select all correct and number them in the order of priority)
7. Pre-school children
8. Primary school children
9. Secondary school children
10. College or university students

THIS IS THE END OF THE QUESTIONNAIRE. THANK YOU VERY MUCH FOR YOUR KIND COOPERATION
